# Automated body weight measurement using computed tomography

**DOI:** 10.1038/s41598-025-24060-1

**Published:** 2025-10-15

**Authors:** Sebastian Schenkl, Holger Muggenthaler, Rahel Koch, Andreas Heinrich

**Affiliations:** 1 Institute of Forensic Medicine, Jena University Hospital – Friedrich Schiller University, Am Klinikum 1, 07747 Jena, Germany; 2 Department of Radiology, Jena University Hospital – Friedrich Schiller University, Am Klinikum 1, 07747 Jena, Germany

**Keywords:** Computed tomography, Body weights and measures, Body composition, Forensic medicine, Computer-assisted image processing, Computed tomography, Anatomy

## Abstract

Computed tomography (CT) images provide detailed information on tissue composition and density. Body weight estimation from CT can be highly relevant in emergency and forensic contexts where conventional weighing is impractical or unavailable—for example, due to unconscious or immobilized patients, lack of appropriate equipment, or the necessity to preserve forensic trace evidence. An automated method for body weight estimation using antemortem and postmortem CT data from clinical scanners was evaluated. Two retrospective cohorts were included: 30 forensic postmortem whole-body CT scans and 66 clinical torso CT scans. Tissue segmentation applied predefined Hounsfield Unit thresholds for fat, muscle, and bone, with voxel volumes multiplied by corresponding tissue densities to calculate weight. In forensic cases, CT-derived weights differed by − 1.26 ± 2.58 kg (median: − 1.15 kg) from measured weights. In clinical cases, torso weight was extrapolated to total body weight using a corrected scaling factor that accounted for body and scan length, resulting in an average difference of 0.53 ± 4.62 kg (median: 0.23 kg). Linear regression demonstrated strong correlations (R^2^ = 0.97/0.96 forensic/clinical). These findings indicate that routinely acquired CT scans enable accurate, automated, and retrospective body weight estimation, providing a non-invasive alternative when direct measurement is not feasible while simultaneously allowing body composition analysis.

**Supplementary Information:**

The online version contains supplementary material available at 10.1038/s41598-025-24060-1.

## Introduction

In clinical practice, unknown patients often present significant challenges, particularly in trauma centers and emergency facilities handling cases from severe accidents, natural disasters, terrorist attacks, multiple injuries, migration, or homelessness^1–3^. In emergency medicine, appearance-based estimation of body weight is highly fraught with uncertainties especially in cases with unconscious patients not being able to provide any self-estimations^4^. Direct body weight measurement can often be impractical or unavailable in these settings, for example when patients are unconscious, severely injured, immobilized on vacuum mattresses, or when immediate transfer to a scale would delay critical treatment. In forensic cases, preserving traces and the state of evidence frequently prohibits any manipulation or movement of the body, preventing direct weight measurement, whereas CT scanning can be performed without disturbing the body or compromising trace evidence. Body mass is not only a crucial parameter for therapy planning and medication dosing but also plays a significant role in identification processes, enabling faster identification through body weight matching in large databases. Computer vision (CV)-based personal identification^5–8^ facilitates the automated matching of recent radiological images with clinical databases, enabling the identification of unknown individuals. When body weight is known, this process can be further expedited by narrowing the search to the most relevant database entries. In forensic investigations, body mass is essential for temperature-based time of death estimation methods and for biomechanical reconstructions, particularly in cases involving violent crimes such as stomping, kicking and punching. For instance, in stomping higher body weight was related to higher contact forces^9^. However, body weight estimation is complicated by factors such as clothing and postmortem evaporation-induced weight loss^10^. Furthermore, weight measurement might change the state of evidence, though preserving traces is essential in the forensic context. Despite these challenges, body weight remains a fundamental parameter especially for empirical and physical models used in forensic death time estimation. Models such as those by Henßge and Madea^11^ and Mall and Eisenmenger^12^ rely heavily on body weight measurements or accurate weight estimations. Errors in weight estimation can lead to significant inaccuracies in determining the time of death, as shown by studies on temperature-based postmortem interval estimations^13^.

To estimate body weight from CT, several methods have been proposed. Ichikawa, et al.^14^ employed deep learning on CT chest and abdominal scout images prior to the CT scan in order to determine contrast medium dose, achieving high accuracy. Gascho et al.^15^ utilized CT dose modulation data by using CT images from decedents. In their study, decomposition changes over extended postmortem intervals led to less accurate body weight estimates. For forensic scenarios, Jackowski et al.^16^ performed a threshold based whole body segmentation without differentiating tissue types. They fitted a multiplication factor in terms of the mean overall body density. However, these methods often require advanced computational tools or are limited to specific contexts. A straightforward yet precise method utilizing CT images from a single scan would significantly simplify the process. However, currently there is no method applicable to both forensic and clinical practice without requiring specialized software or extensive post-processing.

This study aims to develop and evaluate a simple, cost-effective workflow for estimating body weight using routine CT data. The approach integrates segmentation of bone, muscle, and fat volumes, along with automatic removal of the CT table. Additionally, the study aims to validate whole-body weight estimation from CT scans by utilizing forensic postmortem whole-body datasets and antemortem torso CT scans, with the goal of extrapolating the torso mass to the total body weight.

## Methods

This study was approved by the institutional review board (IRB) at Jena University Hospital (registration number 2019–1505-MV) and was carried out in accordance with relevant guidelines and regulations. Due to the retrospective nature of the investigation, the need for written informed consent was waived by the IRB at Jena University Hospital.

### Cohorts

This retrospective study included two distinct cohorts. The first cohort consisted of forensic postmortem cases (whole body), while the second cohort comprised clinical cases from routine imaging of the torso. The scanning dimensions in the clinical cohort were based on literature guidelines for trunk and abdomen CT scans but varied slightly due to the clinical setting and medical indications^17,18^. The scanning boundaries are primarily defined from the seventh thoracic vertebra to the lower edge of the pubic ramus. Both cohorts were analyzed to evaluate the accuracy and reliability of body weight estimations derived from CT scans, comparing different methods and their applicability in both forensic and clinical contexts.

#### Forensic

A total of 30 whole-body postmortem CT examinations were conducted between October 2012 and October 2024 using the following scanners: 27 × GE LightSpeed VCT, 2 × GE Revolution EVO, and 1 × GE Revolution. Voltage settings were 29 scans at 120 kVp and 1 scan at 140 kVp; current was 381.17 ± 157.76 mA. Slice thickness and spacing were 24 scans at 0.625/0.625 mm, 2 scans at 2.5/2.5 mm, and 4 scans with varying combinations (0.625/3 mm, 0.625/3.75 mm, 1.25/3 mm, and 3.75/3 mm). All CT data were reconstructed using the “standard” kernel to ensure consistency across cases, as different reconstruction kernels can affect body composition measurements, particularly bone volume and, consequently, weight estimation. The cohort consisted of 30 individuals aged 25 to 89 years (mean age: 61.31 ± 19.71 years; sex distribution: 12 females and 18 males). During the examinations, the arms were positioned either alongside or placed on top of the abdomen. Reference body weight measurements were taken within 24 h postmortem using a calibrated elevation and transportation system (Funeralia GmbH, Würzburg, Germany) with a weight deviation range of ± 0.25%. Clothing and personal items were removed before weighing. Corpses were stored in a cooling chamber at + 4 °C until the weighing procedure.

#### Clinical

A total of 66 CT examinations of the torso to pelvis were performed on living individuals between April 2021 and May 2021 using a GE Revolution scanner. Scanning parameters included 120 kVp for 59 scans and 100 kVp for 7 scans, with a current of 224.91 ± 102.93 mA and a slice thickness and spacing of 0.625 mm. All CT data were reconstructed using the “standard” kernel to ensure consistency across cases. The cohort consisted of 66 individuals (34 females, 32 males) with a mean age of 64.12 ± 12.06 years. During the scans, the arms were consistently positioned above the head. Reference body weight values were obtained from the clinical patient database.

### CT-based estimation of body weight

**Fig. 1:**
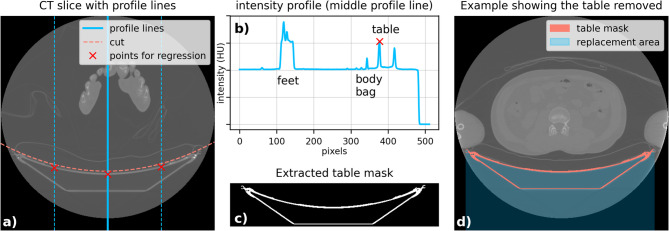
Automatic removal of the CT table: (**a**) Profile lines were drawn at 25%, 50%, and 75% of the image width. (**b**) The intensity profile was analyzed to find the table edges. (**c**) Quadratic regression was used to isolate the table, and (**d**) a mask replaced the table area with − 1000 HU (air).

For the automated CT-based body weight estimation, all CT slices were processed using a three-step approach. First, to exclude the CT table from the images, a desk mask was created by analyzing vertical intensity profiles at 25%, 50%, and 75% of the image width. These profiles were smoothed using Gaussian filtering, followed by peak detection to identify the edges of the table. A quadratic regression was applied to these points to accurately isolate and remove the table region, which was then replaced with − 1000 HU, representing air (see Fig. 1). An alternative method for CT table removal was tested on a subset of 14 whole-body postmortem CT examinations, where nearly the entire body was imaged. This workflow required converting Digital Imaging and Communications in Medicine (DICOM) files into Neuroimaging Informatics Technology Initiative (NIfTI) format before applying TotalSegmentator^19^ (v2.10.0) in “body” mode to generate a 3D whole-body mask, allowing automatic exclusion of non-body voxels, including the CT table. While this approach can improve segmentation accuracy under ideal imaging conditions, it involves additional conversion steps and longer processing times.

Next, tissue segmentation was performed using predefined Hounsfield Unit (HU) thresholds for different tissue types. Fat was segmented between − 190 and − 30 HU^20–24^, muscle between − 31 and 150 HU^20–22,24–26^, and bone above 150 HU. These thresholds were selected based on the comprehensive review by Engelke et al.^27^, which summarizes current standards for CT-based quantitative analysis of skeletal muscle. Additionally, small variations in these thresholds at the fat-muscle and muscle-bone boundaries have a negligible impact on estimated body weight, as demonstrated in Supplementary Figs. S1 and S2. Similarly, shifting the lower threshold for fat segmentation (− 190 HU) or introducing an upper threshold for bone segmentation has a negligible impact on estimated body weight, as shown in Supplementary Figs. S3 and S4. For each slice, masks were created to represent the spatial distribution of each tissue type. Finally, volume and weight estimation were carried out by calculating voxel volumes based on pixel spacing, slice spacing, and slice thickness information obtained from the DICOM files. The tissue volumes were then multiplied by their respective densities—fat at 0.94 g/cm^3^^3,28–30^, muscle at 1.06 g/cm^3^^3,31,32^, and bone at 1.85 g/cm^3^^3,33,34^—to estimate the total body weight. These tissue densities are commonly used standard values derived from cadaver studies and imaging-based measurements and represent average values across different individuals. Small variations in these densities have only a minor impact on estimated body weight, as demonstrated in Supplementary Figs. S5, S6, and S7.

### Evaluation

CT-based body weight estimation was performed for both forensic and clinical cohorts. In the forensic cohort, whole-body CT scans were used to estimate body weight, and these estimations were directly compared with actual forensic weight measurements for validation. In the clinical cohort, where the patients underwent just a torso CT scan, extrapolation methods using scaling factors sf were applied to estimate total body weight from torso weight.

$$total\_body\_weight = \frac{torso\_weight}{{{\text{sf}}}}$$

The scaling factors for estimating whole-body weight from clinical torso CT scans were determined by calculating the ratio of the estimated torso weight to the actual total body weight. These scaling factors were determined as the mean ratios across all subjects and separately for males and females. The overall scaling factor sf was 0.4182, while the values for males and females were 0.4222 and 0.4144, respectively.

The computation of the corrective scaling factor csf utilizes the known whole body length and the length of the torso scan in terms of a normalization to the true relative torso length. The torso scan length was determined by multiplying the slice numbers with the slice thickness.

$$csf = {\text{sf}} + \left( {\frac{scan\_length}{{body\_length}} - x} \right)$$

The offset x = 0.269 was determined by variation of x, minimizing the mean difference between the estimated body weights and the true body weights of the clinical cases.

## Results

The following section provides the results of our weight estimation workflow based on the 30 whole-body postmortem CT scans and the 66 torso-to-pelvis CT examinations.

### Forensic dataset

**Fig. 2:**
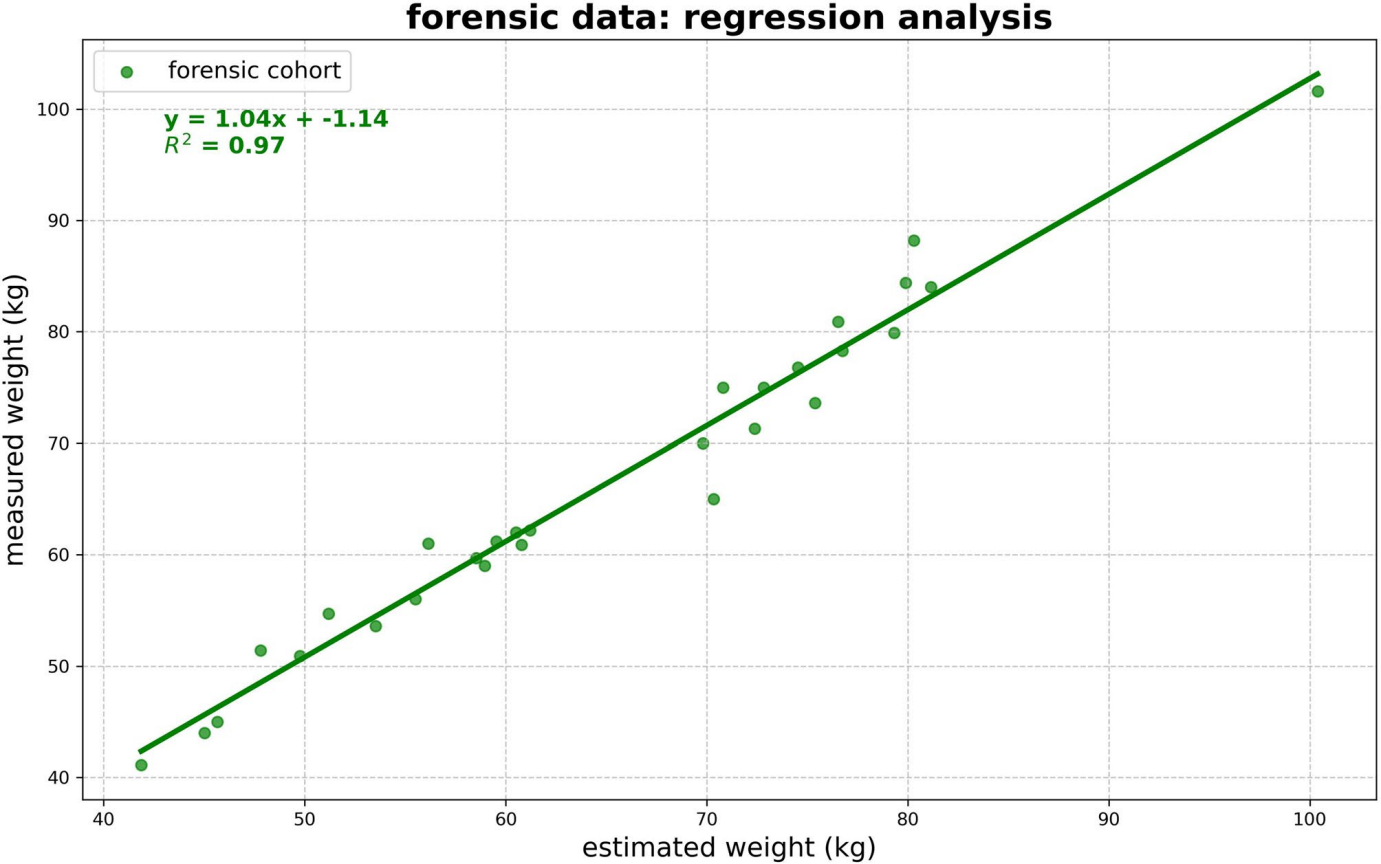
The figure illustrates the relationship between CT-based whole-body weight estimation and forensic measured weight for all subjects, males, and females. A linear regression line is included in each plot to highlight the correlation between these two variables.

**Fig. 3:**
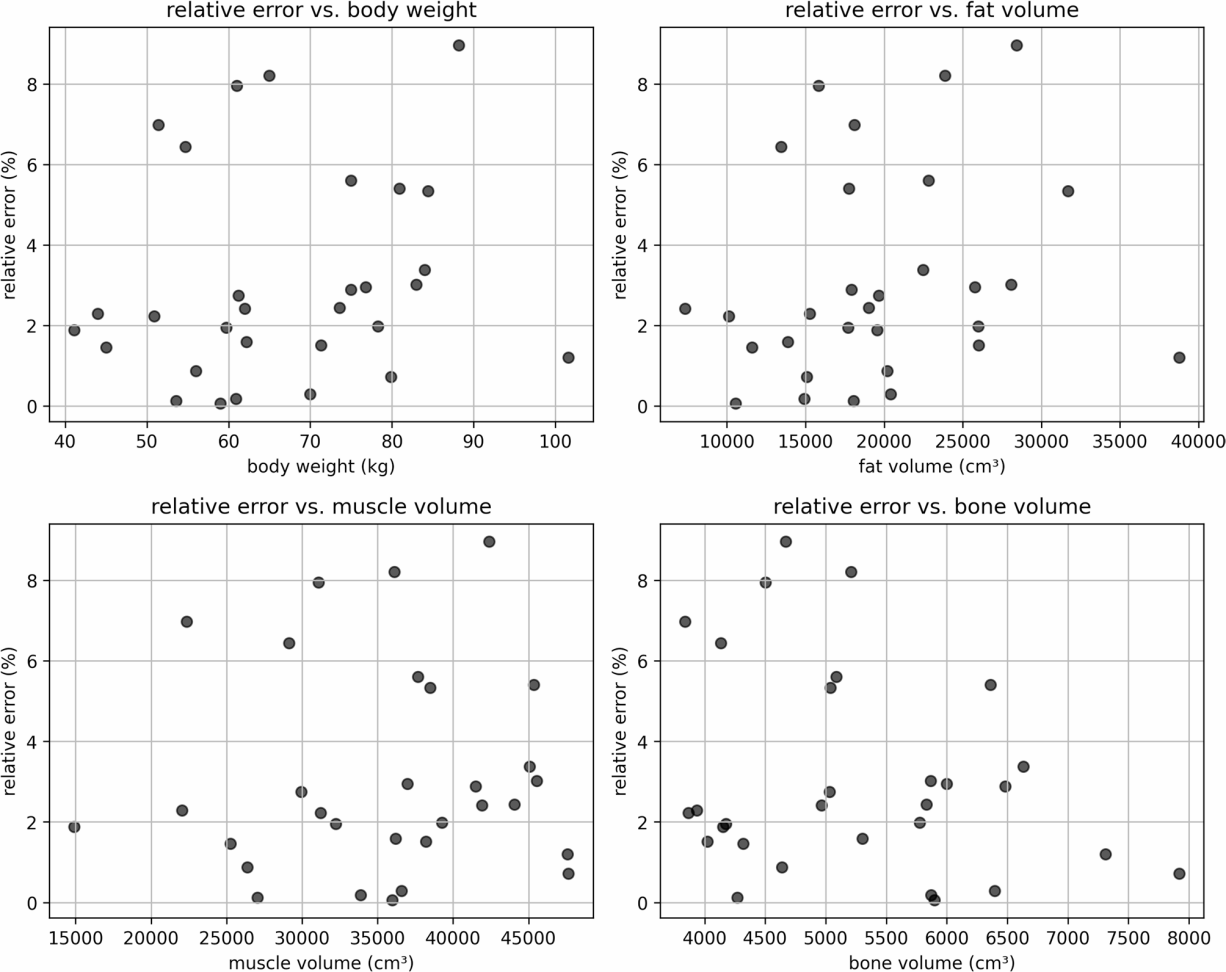
Relative error of the body weight estimation plotted against body weight (top left), fat volume (top right), muscle volume (bottom left), and bone volume (bottom right) for all individuals in the forensic dataset. No systematic correlation was observed between relative error and any of these parameters, indicating robustness of the method across a wide range of body compositions.

For the forensic dataset, the average (negative) difference between measured weight and CT-derived estimations was − 1.26 ± 2.58 kg (median: − 1.15 kg), with an absolute deviation of 2.14 ± 1.89 kg (median: 1.52 kg). Linear regression demonstrated high accuracy, with an R^2^ value of 0.97 (see Fig. 2 and Table 1). The median relative deviation is the same for males and females; however, the absolute weight estimation is more accurate for females. The mean relative error was 3.10 ± 2.56% (see Fig. 3). Correlation analysis showed no relevant relationship between the relative error and body weight (r = 0.16), fat volume (r = 0.20), muscle volume (r = 0.04), or bone volume (r = − 0.25). These results indicate that the method is robust across a wide range of body compositions and does not systematically over- or underestimate body weight in this forensic cohort. The highest relative deviation was observed in the case with a slice thickness and spacing of 3.75 mm and 3 mm. Although the voxel-based volume calculation remains accurate in this setting, a slice thickness larger than the slice spacing can lead to partial volume effects and HU interpolation artifacts, which may slightly affect density-based weight estimation. This limitation could not be corrected retrospectively.

**Table 1:** Summarizes the differences between CT-derived weight estimations and reference measurements for both the forensic (postmortem) and clinical cohorts, with results further stratified by sex.

Sex	Difference CT-derived estimations and reference					R^2^
	MED relative deviation (%)	Mean (kg)	MED (kg)	Abs. mean (kg)	Abs. MED (kg)	
Forensic (postmortem)						
All	2	− 1.26 ± 2.58	− 1.15	2.14 ± 1.89	1.52	0.97
Male	2	− 0.96 ± 2.54	− 1.19	2.15 ± 1.59	1.67	0.95
Female	2	− 1.71 ± 2.68	− 0.81	2.12 ± 2.35	1.07	0.99
Clinical (whole body)						
All	6	0.71 ± 6.38	− 0.56	5.20 ± 3.71	4.52	0.95
Male	6	0.55 ± 6.64	1.24	5.39 ± 3.80	4.75	0.93
Female	6	0.77 ± 6.17	− 0.76	4.90 ± 3.72	3.97	0.96
Clinical (whole body normalized by scan length and body height)						
All	3	0.53 ± 4.62	0.23	3.54 ± 2.98	2.51	0.96
Male	4	0.38 ± 5.09	1.05	3.95 ± 3.16	3.10	0.95
Female	4	0.57 ± 4.11	0.76	3.18 ± 2.60	2.48	0.98

Reported values include the median (MED) relative deviation as a percentage, mean and median differences, absolute mean and median deviations, and the R^2^ value for each cohort. The clinical cohort is categorized by estimation method for whole-body weight estimates using scale-factor estimation from the trunk CT, and whole-body estimates adjusted for scan length and body height (normalized approach). Scaling factor(s) sf—overall: 0.4182, m: 0.4222; f: 0.4144

**Fig. 4:**
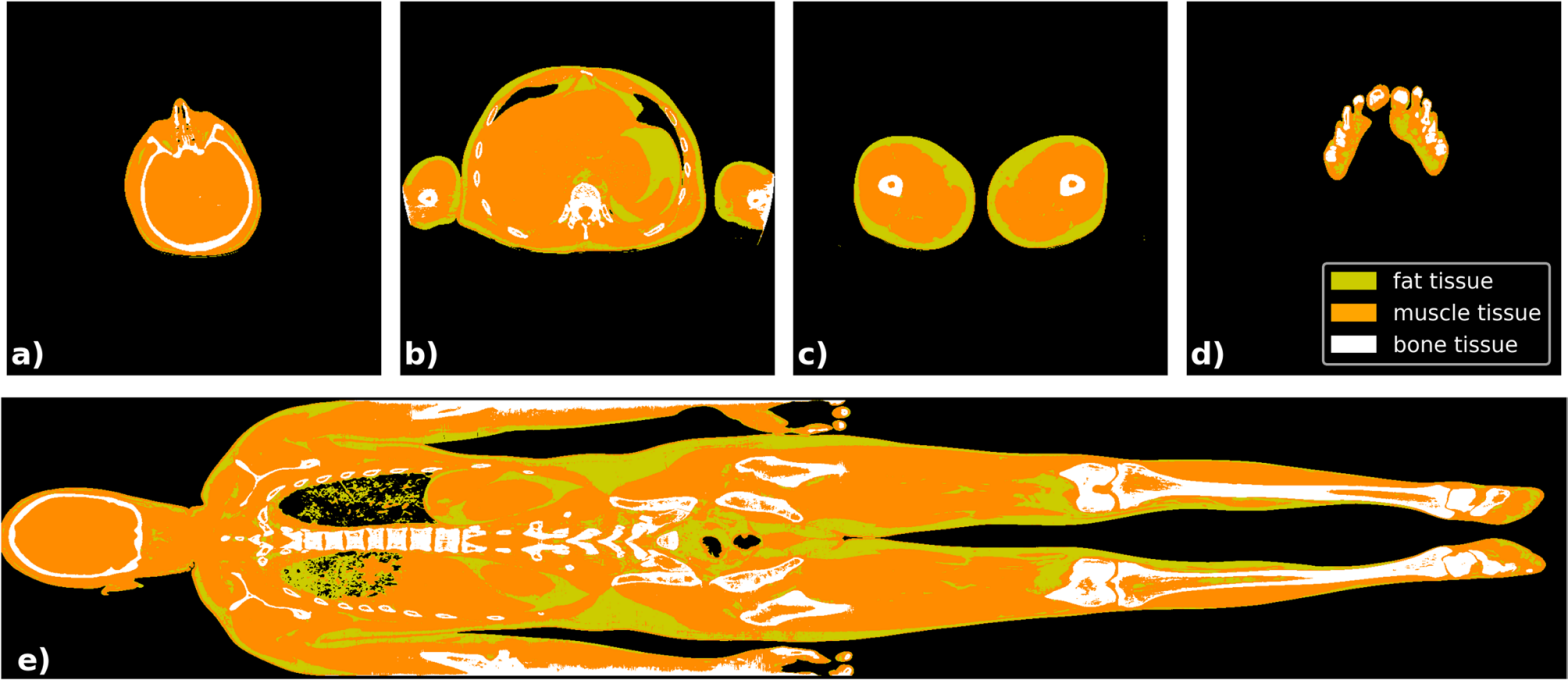
An exemplary visualization of the differentiation between fat, muscle, and bone tissue through thresholding, shown in transverse (**a**–**d**) and coronal (**e**) slices of the whole-body postmortem CT images. If the field of view of the CT scanner is insufficient, sections such as the arms may be partially cut off in the CT images.

Further analysis of the cohort revealed that in 16 out of 30 cases, certain parts of the body were to some extent outside the field of view of the CT scanner (see Fig. 4), resulting in under-representation of body volume. For the remaining 14 cases with nearly fully imaged bodies, the mean deviation from the reference weight was significantly reduced to 0.32 ± 2.22 kg (median: 0.31 kg), with an absolute deviation of 1.56 ± 1.55 kg (median: 1.04 kg), highlighting the reliability and precision of the developed workflow under optimal imaging conditions. Comparison with TotalSegmentator-based body masks showed comparable results (mean deviation: − 0.02 ± 2.23 kg, median: 0.02 kg; absolute deviation: 1.60 ± 1.49 kg, median: 0.92 kg). Without removal of the CT table, the mean and absolute deviation from the reference weight were identical at 5.70 ± 2.27 kg (median: 5.48 kg).

### Clinical dataset

**Fig. 5:**
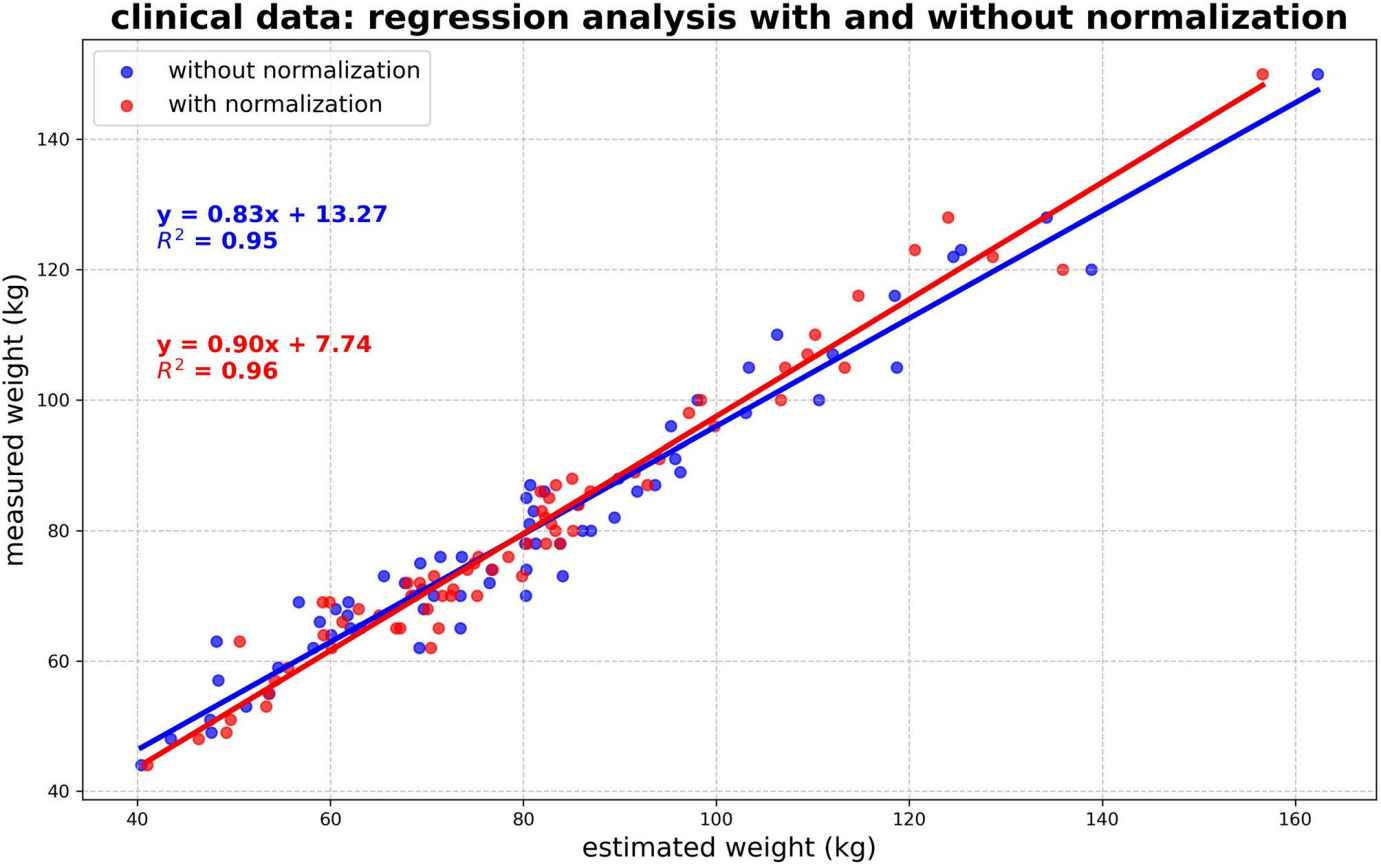
The figure illustrates the relationship between CT-based whole-body weight estimation (with torso weight scaled by the factor sf (blue) and normalized by scan length and body height using csf (red) and measured weight for all subjects, males, and females. Linear regression lines are included to highlight the correlation between the variables.

**Fig. 6:**
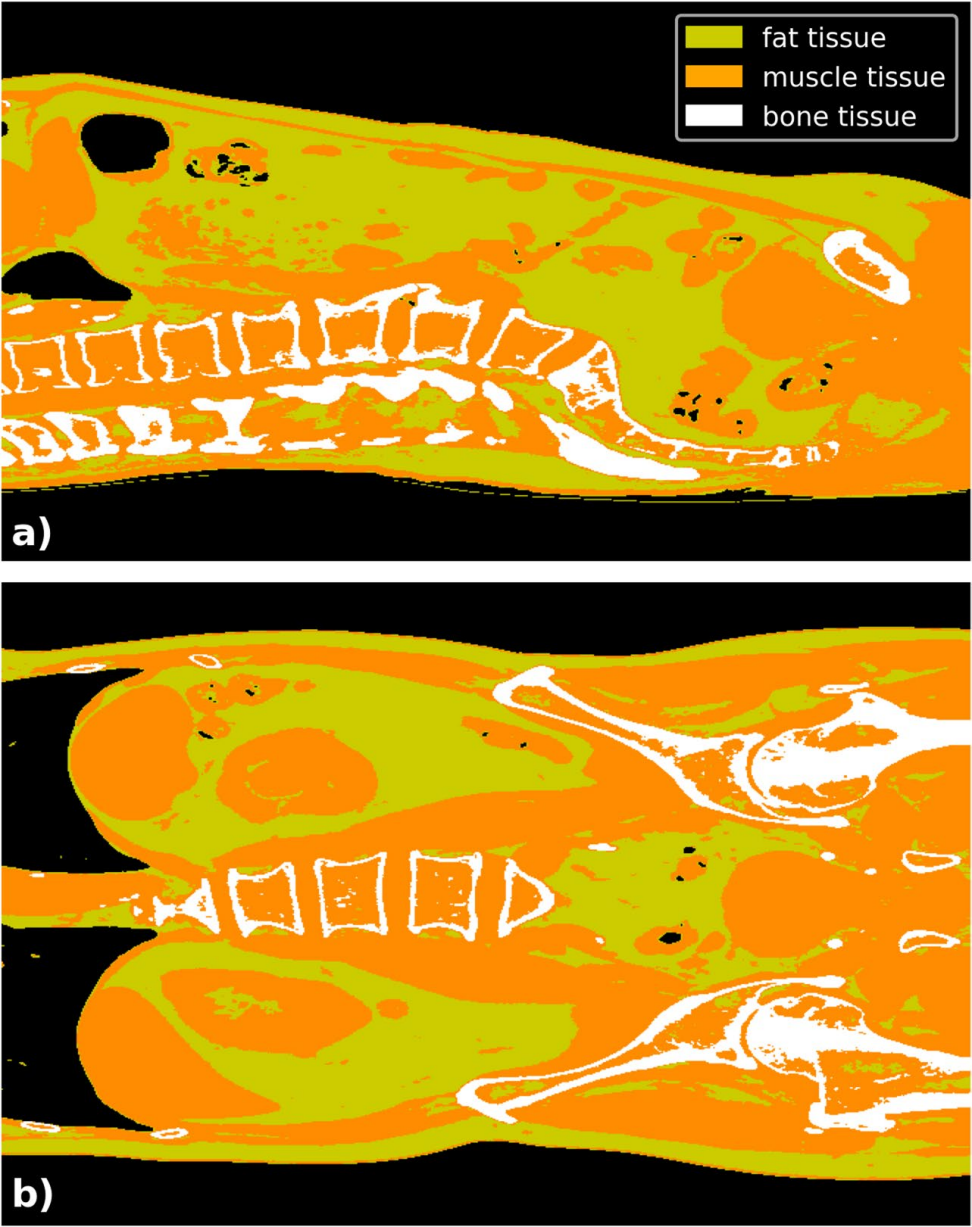
An exemplary visualization of the differentiation between fat, muscle, and bone tissue through thresholding, shown in sagittal (**a**) and coronal (**b**) slices of clinical torso-to-pelvis CT images, where the arms were positioned above the head.

**Fig. 7:**
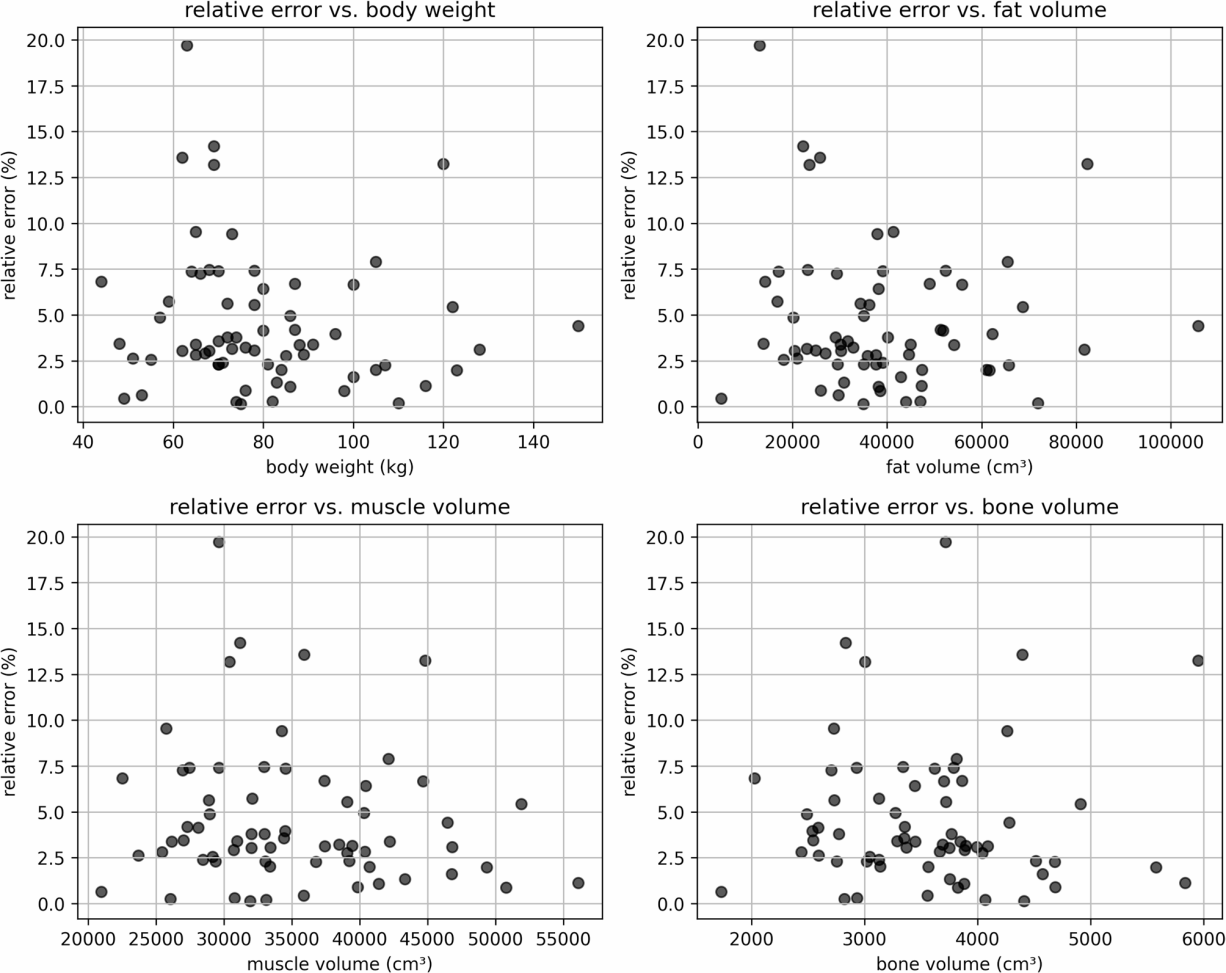
Relative error of the body weight estimation plotted against body weight (top left), fat volume (top right), muscle volume (bottom left), and bone volume (bottom right) for all individuals in the clinical dataset. No systematic correlation was observed between relative error and any of these parameters, indicating robustness of the method across a wide range of body compositions.

The clinical cohort, taking torso CT-scan into account, was analyzed for weight estimation quality in two different ways using a scaling factor sf and a corrected scaling factor csf. The calculation of the overall scaling factor sf—representing the ratio of total body mass to trunk mass—yielded a value of 0.4182, with sex-specific values of 0.4222 for males and 0.4144 for females, respectively. The body weight estimation under application of sf reveals an average difference of 0.71 ± 6.38 kg (median: -0.56 kg), with an absolute deviation of 5.20 ± 3.71 kg (median: 4.52 kg) regarding the measured whole body weight. The mean deviation between estimated and measured body weight was 0.53 ± 4.62 kg (median: 0.23 kg) using the corrected scaling factor csf. Linear regression demonstrated high accuracy, with an R^2^ value of 0.95 for the whole-body dataset through the use of sf, and 0.96 after applying csf (see Fig. 5). The median relative deviation is the same for males and females; however, the absolute weight estimation is more accurate for females, with a lower absolute deviation (see Table 1). Figure 6 shows an example of the segmentation of fat, muscle, and bone. The mean relative error across all cases was 4.54 ± 3.80% (see Fig. 7). Importantly, the relative error showed no relevant correlation with body weight (r = − 0.12), fat volume (r = − 0.10), muscle volume (r = − 0.12), or bone volume (r = − 0.00), indicating that the method does not systematically over- or underestimate body weight in lighter individuals and is robust across a wide range of body weights.

## Discussion

This study demonstrates the feasibility of estimating total body weight from CT scans acquired with clinical CT scanners, offering a rapid and practical approach suitable for both forensic and clinical contexts. The strong linear correlation between measured and CT-estimated body weight, with deviations of only a few kilograms, underscores the accuracy of the developed method. Variations in segmentation thresholds and tissue density assumptions had a negligible impact on the accuracy of the body weight estimates, highlighting the robustness of the proposed method despite potential variability in image processing and biological tissue properties. Forensic and clinical application must be considered separately. The forensic cohort achieved weight estimations with deviations ranging from 1 to 2 kg compared to measured body weight, performing on par with or exceeding the accuracy of existing methods. However, incomplete imaging data in the forensic cohort substantially impacted accuracy, with discrepancies reaching up to − 4.85 kg in cases where body parts were missing or truncated in CT scans. An even larger discrepancy of − 7.91 kg was observed in a case where missing body parts coincided with a slice thickness exceeding the slice spacing. This combination led to partial volume effects and HU smoothing, further contributing to the underestimation in density-based weight calculation. In contrast, fully imaged postmortem bodies exhibited minimal deviations (e.g., 0.04 kg), emphasizing the necessity of complete imaging for precise weight estimation.

Several open-source tools, such as 3D Slicer^35^, provide functionalities for CT table removal, though these typically require user interaction or custom scripting for robust automation. Automated segmentation tools like TotalSegmentator provide predefined whole-body masks that automatically exclude the CT table and foreign objects. While this workflow standardizes analysis pipelines, it requires prior data conversion, longer processing times, and does not directly segment generic tissue classes such as fat, muscle, and bone across the entire volume. TotalSegmentator offers a “body” mode that generates comprehensive masks including skin and subcutaneous fat, whereas the “total” mode combines organ and structure segmentations but may leave gaps in diffuse soft tissues, limiting its completeness for body composition analysis. By comparison, the desk mask method operates directly on original DICOM slices and enables CT table removal on a per-slice basis without format conversion or manual interaction. This allows flexible handling of datasets with varying resolutions or partial body segments—such as separately scanned head and torso images—that cannot be easily combined using TotalSegmentator, offering a streamlined and adaptable workflow particularly suited for heterogeneous or fragmented datasets.

In this retrospective dataset, incomplete scan coverage frequently resulted from arms or parts of the feet extending beyond the field of view, reflecting the real-world variability in postmortem CT acquisitions. Potential improvements could include scanning the body in multiple overlapping parts (e.g., left and right body halves) and subsequently merging these datasets to achieve full-body coverage. Methods adapted from photographic stitching could assist in automatically identifying and aligning overlapping regions. However, this approach may be challenging in cases of high BMI, rigor mortis, or scanner limitations and should be explored in future prospective studies. In parallel, estimation methods incorporating factors such as sex, age, average bone mass, and trunk and limb fat-to-muscle ratios could be developed to approximate the weight of missing segments when full-body coverage cannot be achieved.

Compared to existing methods, the proposed technique demonstrates comparable or smaller errors relative to published approaches^14–16^. The existing literature highlights various approaches to CT-based weight estimation, such as deep learning on scout images^14^ and CT dose modulation data^15^. These methods often require advanced computational tools or are tailored to specific contexts. In contrast, the method developed here leverages standard clinical CT data without complex post-processing or additional algorithms, making it accessible to clinicians and coroners.

For partial-body scans of the clinical trunk CT-data, the accuracy of body weight estimation depended on the chosen method. In addition to the scaling factor sf, which represents the relative proportion of trunk mass to total body mass, the corrective scaling factor csf was determined to normalize the length and height of the trunk scan, thereby improving the accuracy of the results. Total body height can be obtained from the clinical CT-sets or from the CT scout image. These findings indicate that CT-based weight estimation can serve as a valuable tool in clinical settings, particularly in scenarios where direct body weight measurement is impractical or unavailable, such as for specific patient populations or in emergency situations.

Although manual direct weighing with scales is usually the fastest and simplest method to obtain body weight, many clinical and forensic scenarios make it impractical or impossible. For example, unconscious or severely injured patients are often immobilized on stretchers or vacuum mattresses, making transfer to a scale unsafe or harmful. In forensic cases, moving the body to weigh it can compromise trace evidence or alter its condition, which is unacceptable. In such situations, CT scanning—often already part of routine workflows—provides a non-invasive, contactless alternative. Additionally, CT can provide relevant information on the depth of rectal thermometer insertion, improving postmortem interval estimation accuracy. While CT acquisition and postprocessing require time, these can be integrated seamlessly into existing protocols without delaying urgent treatment or investigation. Modern CT scanners enable rapid imaging, and the automated postprocessing here allows fast, objective weight estimation without additional patient handling. Moreover, CT-based weight estimation simultaneously delivers detailed body composition data (e.g., fat, muscle, bone volumes) unavailable from simple weighing. This dual benefit enhances therapy planning, medication dosing, and forensic analysis beyond mere weight determination. Thus, the approach offers an efficient, precise alternative when direct weighing is unfeasible, combining speed, safety, and additional diagnostic information in one examination.

The accuracy of the whole-body weight estimations depends on the chosen scaling factor as well. The literature provides a percentage of 41.6% of the trunk volume with respect to the whole body weight in Pearsall et al.^18^. This value is quite close to our findings with an overall mean scaling factor of 0.4182 and sex specific values of 0.422 for males and 0.414 for females. However, it is evident that the exact proportion of trunk volume is influenced by the length of the scanned body region. The corrected scaling factor approach, which incorporates scan length, helps mitigate this source of uncertainty. Applying the normalization approach reduced the body weight estimation error in the clinical cohort from approximately 6% to 4%. With the aim of achieving further optimization, the scaling factor and the corrected scaling factor should be refined leveraging a larger pool of data. Future investigations should explore the integration of CT scout images, as they may offer additional information regarding the relative scan length of the individual torso section. Such data could allow for more precise, personalized normalization in estimating body weight from partial-body CT scans as done here to a certain degree through the use of the corrected scaling factor.

Analysis across both cohorts confirmed the feasibility of CT-based body weight estimation and identified potential for further improvements to enhance precision. The forensic dataset, with its full-body scans, exhibited lower standard deviations compared to the clinical cohort, highlighting the superiority of complete compared to partial torso CT scans. Conversely, the clinical dataset demonstrated the practicality of a correlation-based approach for rapid weight estimation.

Sex-specific discrepancies in the accuracy of weight estimation were observed when evaluating the results. Higher in females may be explained by their narrower weight range and lower variability in body composition. There are pronounced sex-specific differences in visceral fat distribution. Men generally possess both a higher absolute and relative proportion of visceral fat compared to subcutaneous fat than women. The combination of visceral fat, organs, and connective tissue forms a more homogeneous mixed tissue in the trunk region, making it somewhat more challenging to accurately segment fat and muscle tissue. For both sexes, tissue segmentation using HU thresholding for muscle and fat is not entirely precise due to the mixed-pixel issue, particularly in organ and connective tissues^24^.

In major criminal investigations, rapid body weight estimation is critical for identification and determining time of death^24^. When body weight is unknown or uncertain, death time reconstruction based on rectal temperature becomes infeasible. Additionally, CV-based personal identification^5–8^ could benefit from CT-based body weight estimation. When total body weight is objectively estimated, this process can be further accelerated, as only the most relevant database entries—those more likely to match the estimated body weight—need to be examined. However, as body weight can vary substantially within the same individual over time, weight-based filtering should not be considered an absolute exclusion criterion but rather as an initial prioritization step within the identification workflow. If no relevant match is found, the acceptable weight range can be iteratively expanded (e.g., ± 5 kg, then ± 10 kg), similar to how estimated age ranges^5,36^ are widened during searches. Combining objective estimates of weight, age, and sex in this stepwise manner may further enhance the efficiency of database searches while maintaining flexibility in cases of variable body weight, especially in emergency or forensic scenarios where quick identification is crucial.

Although the method is robust, it has some limitations. While the current dataset is reliable, its size remains limited. Missing body parts, e.g. with incomplete arm or leg scans, could affect accuracy in forensic cases, and strategies to estimate their weight would improve results. Additionally, the assumption of uniform body composition and tissue densities may not apply to all populations, as individual variability in body fat, muscle, and particularly bone density can occur, potentially introducing minor inaccuracies. For example, fat density can vary depending on its water content and metabolic state^29^. Although bone density varies across different bones (e.g., between trabecular and cortical bone), most of the skeleton’s weight comes from dense cortical bone. Therefore, this variability has only a minor effect on total body weight estimation. In cases of fragmented remains, using “weight clusters” for extremities and the head could enhance accuracy. Despite these limitations, the method offers a reliable, cost-effective solution for body weight estimation in both clinical and forensic settings. Future improvements will focus on handling incomplete or fragmented bodies.

In conclusion, this study highlights the potential of CT-based body weight estimation as a valuable tool in both clinical and forensic contexts. The method is straightforward and universally applicable using CT-scanners in a “medical routine”. While several CT-based approaches for weight estimation have been proposed, many rely on advanced computational tools, extensive post-processing, or are limited to specific applications.

## Supplementary Information

Below is the link to the electronic supplementary material.

Supplementary Material 1

## Data Availability

The datasets analyzed during the current study are not publicly available due to privacy concerns. However, all relevant data supporting the findings of this study are included in the article. For further inquiries or data requests, please contact the corresponding author.
