# Supplementary material for: Automated body weight measurement using computed tomography
Source: Sci Rep. 2025 Oct 15;15:36110. doi: 10.1038/s41598-025-24060-1 (PMC12528416; doi:10.1038/s41598-025-24060-1)
Supplement: Supplementary file 1 — Supplementary Material 1 [file 41598_2025_24060_MOESM1_ESM.pdf]

Automated Body Weight Measurement Using Computed Tomography

Sebastian Schenkl[1], Holger Muggenthaler[1],
Rahel Koch[2], Andreas Heinrich[2]

[1]Institute of Forensic Medicine, Jena University Hospital –
Friedrich Schiller University, Am Klinikum 1, 07747 Jena, Germany
[2]Department of Radiology, Jena University Hospital –
Friedrich Schiller University, Am Klinikum 1, 07747 Jena, Germany

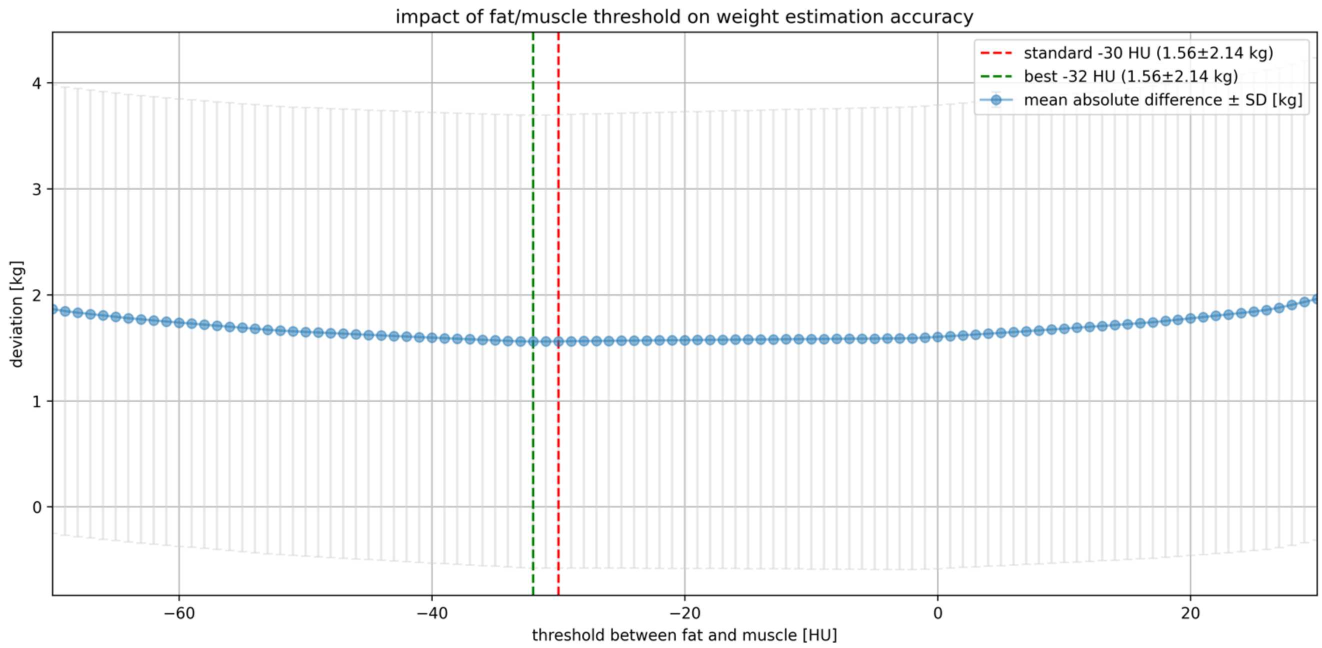

Figure S1 Impact of the HU threshold between fat and muscle on body weight estimation accuracy. The plot shows the mean absolute deviation (± standard deviation) between estimated and measured body weight across different HU thresholds. The dashed red line indicates the applied standard threshold, while the dashed green line marks the threshold with the lowest deviation in a dataset of 14 cases with nearly fully imaged bodies.

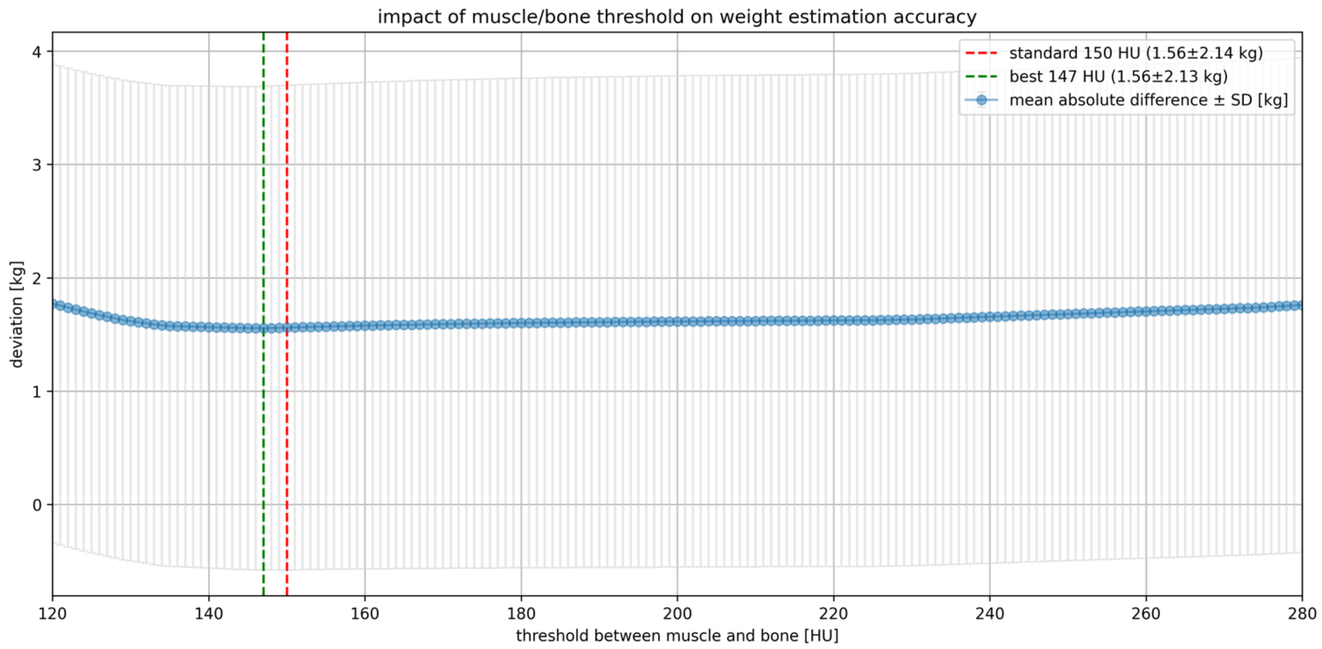

Figure S2 Impact of the HU threshold between muscle and bone on body weight estimation accuracy. The plot shows the mean absolute deviation (± standard deviation) between estimated and measured body weight across different HU thresholds. The dashed red line indicates the applied standard threshold, while the dashed green line marks the threshold with the lowest deviation in a dataset of 14 cases with nearly fully imaged bodies.

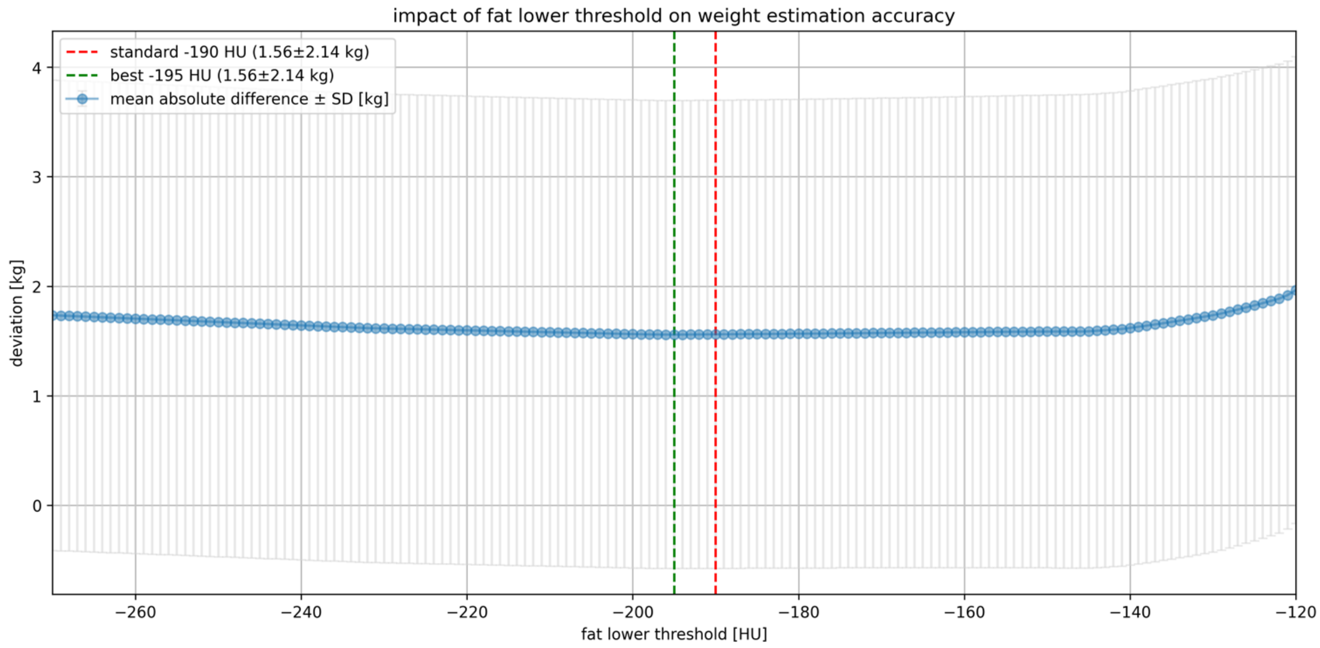

Figure S3 Impact of the HU lower threshold for fat on body weight estimation accuracy. The plot shows the mean absolute deviation (± standard deviation) between estimated and measured body weight across different lower HU thresholds for fat segmentation. The dashed red line indicates the applied standard threshold, while the dashed green line marks the threshold with the lowest deviation in a dataset of 14 cases with nearly fully imaged bodies.

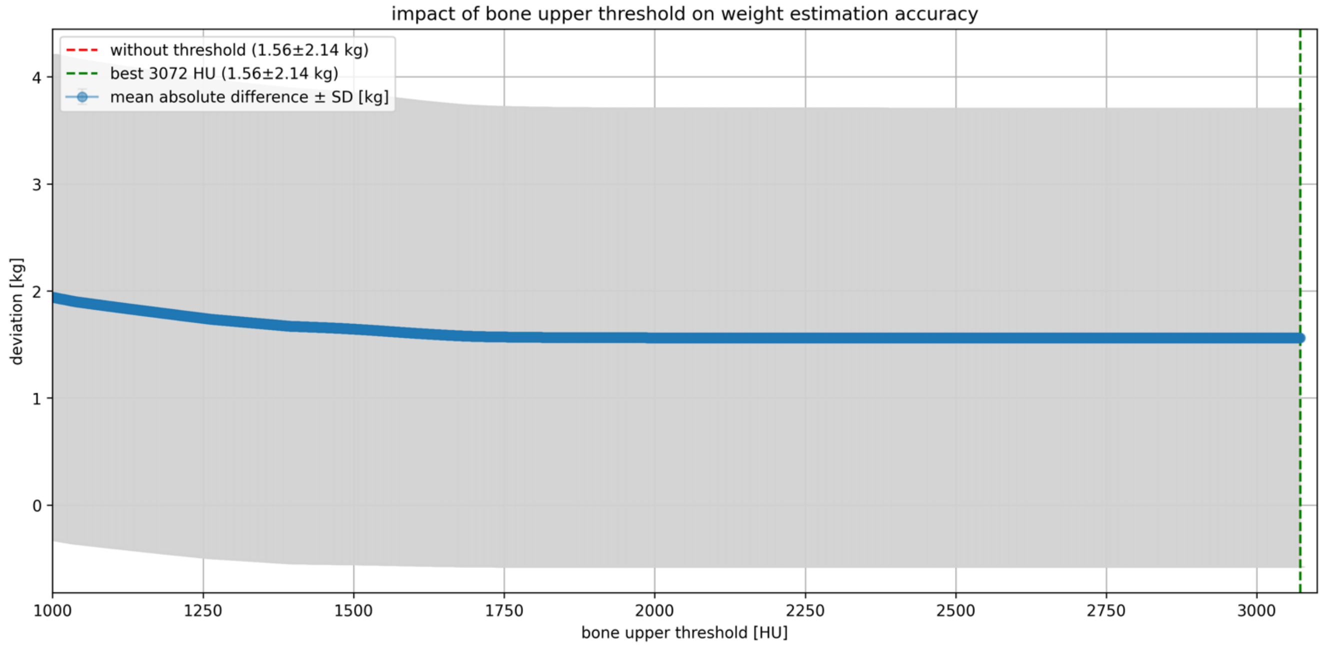

Figure S4 Impact of the HU upper threshold for bone on body weight estimation accuracy. The plot shows the mean absolute deviation (± standard deviation) between estimated and measured body weight across different upper HU thresholds for bone segmentation. The dashed red line indicates the applied setting without an upper threshold, while the dashed green line marks the threshold with the lowest deviation in a dataset of 14 cases with nearly fully imaged bodies.

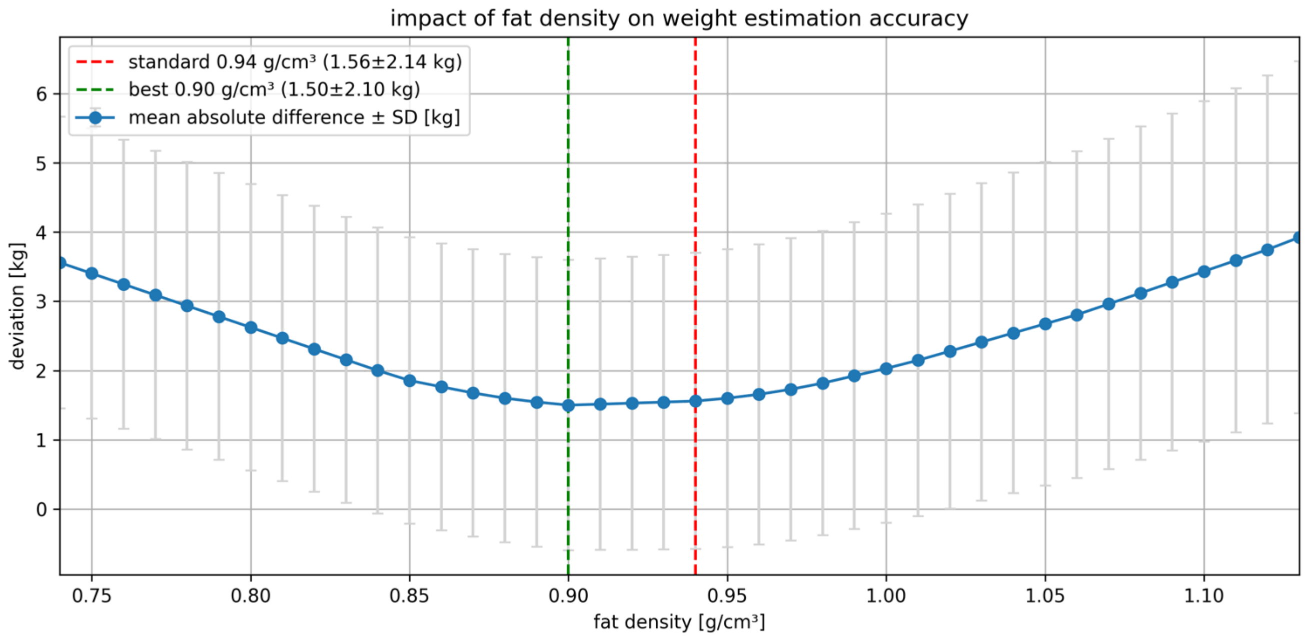

Figure S5 Impact of assumed fat density on body weight estimation accuracy. The plot shows the mean absolute deviation (± standard deviation) between estimated and measured body weight across different assumed fat densities. The dashed red line indicates the applied standard density of 0.94 g/cm³, while the dashed green line marks the density with the lowest deviation in this dataset of 14 nearly fully imaged bodies.

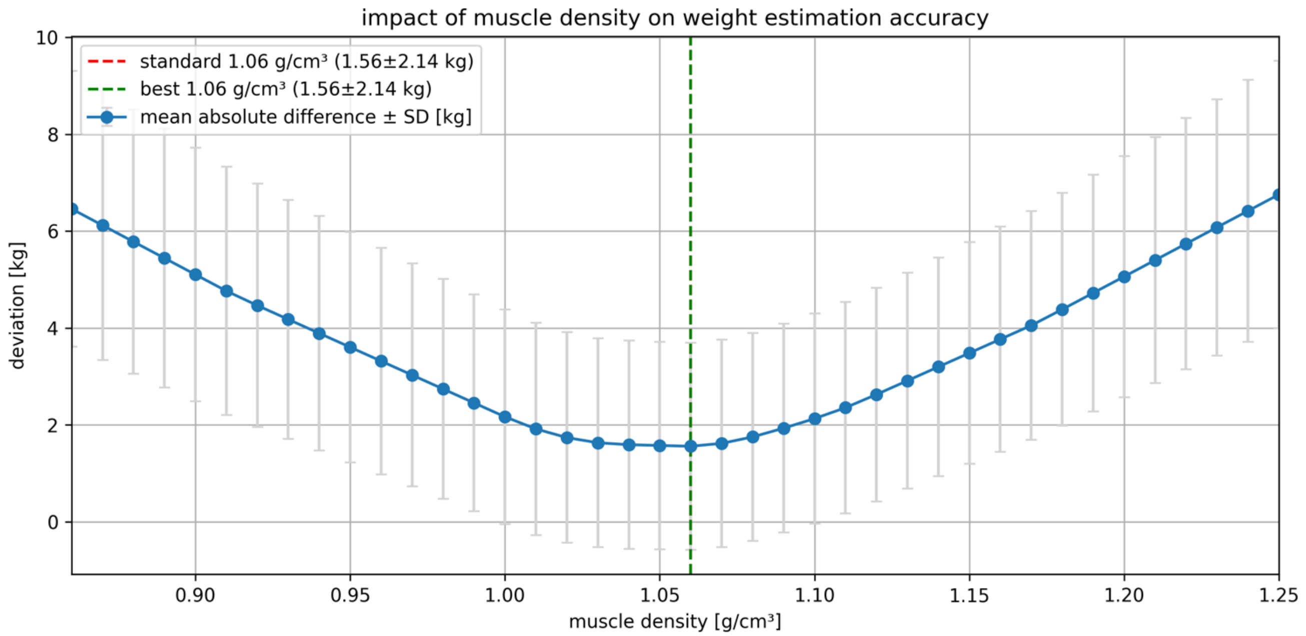

Figure S6 Impact of assumed muscle density on body weight estimation accuracy. The plot shows the mean absolute deviation (± standard deviation) between estimated and measured body weight across different assumed muscle densities. The dashed red line indicates the applied standard density of 1.06 g/cm³, while the dashed green line marks the density with the lowest deviation in this dataset of 14 nearly fully imaged bodies.

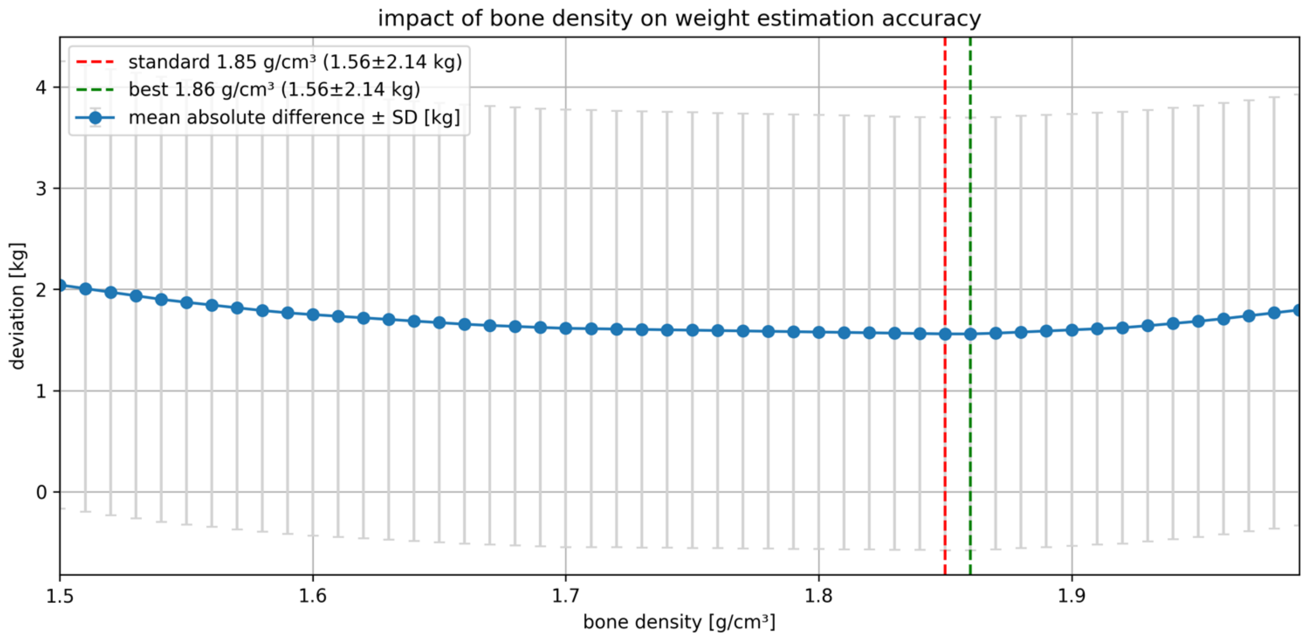

Figure S7 Impact of assumed bone density on body weight estimation accuracy. The plot shows the mean absolute deviation (± standard deviation) between estimated and measured body weight across different assumed bone densities. The dashed red line indicates the applied standard density of 1.85 g/cm³, while the dashed green line marks the density with the lowest deviation in this dataset of 14 nearly fully imaged bodies.
